# Dr. John N. Jones

**Published:** 1911-11

**Authors:** 


Dr. John N. Jones, University of Buffalo 1898, twice coroner of Chemung Co., N. Y., died at Erin, September 5, aged 38.

